# Survey on the perception of germline genome editing among the general public in Japan

**DOI:** 10.1038/s10038-018-0430-2

**Published:** 2018-03-15

**Authors:** Masato Uchiyama, Akiko Nagai, Kaori Muto

**Affiliations:** 1 Department of Computational Biology and Medical Sciences, Graduate School of Frontier Sciences, The University of Tokyo, Kashiwa-shi, Chiba, Japan; 2 Department of Public Policy, The Institute of Medical Sciences, The University of Tokyo, Minato-ku, Tokyo, Japan

**Keywords:** Ethics, Risk factors

## Abstract

Genome editing of human embryos could become a fundamental treatment approach for genetic diseases; however, a few technical and ethical issues need to be resolved before its application in clinical settings. Presently, the Japanese government has issued a statement prohibiting human germline editing and emphasizing the need for discussions that include a wide range of perspectives. However, current discussions tend to exclude the general public. Therefore, we conducted a survey of 10,881 general adults and 1044 patients in Japan who indicated that their disease conditions are related to their genetic makeup, and clarified their attitude toward this technology. The results clearly indicated that the Japanese people generally accepted the use of genome editing for disease-related genes, but many were concerned about the risks. In addition, many Japanese people did not understand the technology well. To improve awareness and understanding about genome editing, it is important that scientists and science communicators create opportunities for the public to participate in relevant discussions without harming vulnerable participants. It is also important to continuously track changes in the acceptance of genome editing by the public.

Genome editing involves insertion, deletion, or modification of DNA with increased specificity and efficiency at a specific site in the genome [1]. This technology can be applied to research, agriculture, and medical care. Following the first application of the CRISPR/Cas9 system in abnormally fertilized embryos by Chinese researchers [2], the United States National Academy of Sciences hosted an international summit and promptly issued a statement regarding serious concerns related to germline genome editing, including risks of inaccurate editing and ethical issues [1]. In Japan, the Expert Panel on Bioethics of the Council for Science, Technology, and Innovation issued a tentative statement on the use of genome editing in human embryos, raising similar concerns including the difficulty of predicting possible harmful effects of genetic changes under various circumstances experienced by the human population and the possibility that permanent enhancements in genetic subsets of the population could exacerbate social inequities or be used coercively [3]. Furthermore, presently, four Japanese academic societies have requested that the government prohibit human germline genome editing [4]. Apart from the interim moratorium on clinical application, these statements emphasize the need for discussions that include various perspectives, i.e., those of patients, their families, and the public.

Previous studies have shown that the public is generally supportive of germline genome editing to cure life-threatening diseases, but not for genetic enhancement [5, 6]. Another survey revealed that the people in the United States want to engage in discussions on genome editing [7]. However, these surveys did not investigate stakeholders separately. In particular, patients with genetic conditions are important stakeholders because they or their offspring are likely to be clinical trial participants and beneficiaries of germline editing [8] or, in the worst case, to be candidate eugenic targets if this technology is misused. Thus, we conducted an online survey in Japan to clarify the attitude of the public and patients diagnosed with or at a risk of developing genetic conditions toward genome editing.

Cross-sectional and anonymous online surveys were conducted by administering the same questionnaire to 44,360 general adults (GAs) in the general Japanese population aged 20–69 years and 6522 Japanese patients aged 20–79 years. The study duration ranged from February to March 2017. GAs were extracted from the survey panel of INTAGE Inc., based on sex, age, and residential area according to the national census data. Japanese patients who visited a hospital or were hospitalized for cancer or cardiovascular, cerebrovascular, or psychiatric diseases within the last year were enrolled. After reading the text explaining the characteristics of genome editing, which included differences between genome editing and genetic recombination, and effects of genome editing of fertilized embryos on the next generation, respondents were questioned about the awareness, level of understanding, criteria, and risks of germline genome editing.

The GAs group included 10,881 respondents (response rate: 24.5%) and the Japanese patient group included 4195 respondents (64.3%). We extracted respondents who indicated that their disease conditions were related to their “genetic makeup” (Pts, *n* = 1044) from the Japanese patient group and compared their attitudes with those of the GAs (Table 1).

**Table 1:** Respondent characteristics and awareness and understanding levels of the “genome editing”

	GAs (*N* = 10,881)				Pts (*N* = 1044)			
	Males (*N* = 5397)		Females (*N* = 5484)		Males (*N* = 658)		Females (*N* = 386)	
	*N*	%	*N*	%	*N*	%	*N*	%
Total	5397	49.6	5484	50.4	658	63.0	386	37.0
*Age groups (years)*								
20–29	823	15.2	843	15.4	10	1.5	39	10.1
30–39	1052	19.5	1039	8.9	40	6.1	97	25.1
40–49	1302	24.1	1289	23.5	133	20.2	119	30.8
50–59	1040	19.3	1061	19.3	194	29.5	89	23.1
60–69	1180	21.9	1252	22.8	185	28.1	31	8.0
70–79	–	–	–	–	96	14.6	11	2.8
*Marital status*								
Unmarried	1863	34.5	1381	25.2	134	20.4	114	29.5
Married	3534	65.5	4103	74.8	524	79.6	272	70.5
*Do you have children?*								
Yes	2665	49.4	2239	40.8	414	62.9	182	47.2
No	2732	50.6	3245	59.2	244	37.1	204	52.8
*Educational background*								
Junior high school	141	2.6	124	2.3	19	2.9	20	5.2
High school	1544	28.6	1888	34.4	183	27.8	136	35.2
Occupational school	681	14.4	1911	34.8	89	13.6	117	30.3
Junior college								
University or graduate school	2934	54.4	1561	28.5	367	55.8	113	29.3
*Awareness level*								
Understand what it means	543	10.1	180	3.3	113	17.2	22	5.7
Have heard of it	1721	31.9	1121	20.4	237	36.0	97	25.1
Have never heard of it	5397	58.1	4183	76.3	308	46.8	267	69.2
*True or false question*								
Correct	511	9.5	338	6.2	105	16.0	34	8.8
Incorrect	883	16.4	389	7.1	134	20.4	46	11.9
Not at all	4003	74.2	4754	86.7	419	63.7	306	79.3

*GAs* general adults, *Pts* patients with disease conditions related to their genetic makeup

Regarding the perception of the term “genome editing,” 6.6% of GAs and 11.5% of Pts responded, “understand what it means,” and 67.2% of GAs and 58.0% of Pts responded that they “have never heard of genome editing” (Table 1). Among respondents who indicated, “I understand what it means,” 24.2% of GAs and 31.0% of Pts incorrectly answered a true or false question on the basic nature of the CRISPR/Cas9 system. Pts were more aware of genome editing than GAs, which might be because Pts were more interested in this technology. This result possibly indicates that awareness in Japan is lower than that in the USA (“a lot” 9%, “not at all” 42%) [6].

Furthermore, we investigated differences in the awareness and acceptance rate and perceived risks of human germline genome editing. The survey respondents were age-matched by excluding those aged ≥70 years from the Pt group. Acceptance rates for “may be performed for disease that shorten a baby’s life” were related to awareness in the Pt and GA groups (*P* < 0.01), and residual analysis indicated that the group that answered “understanding what it means” had significantly higher acceptance than the other groups (Fig. 1). In addition, acceptance rates of “may be performed for disease that require long term care” were related to awareness in the Pt and GA groups (*P* < 0.01), and residual analysis indicated that the group that answered “understanding what it means” had significantly higher acceptance than the other groups (Fig. 1). Thus, the high-awareness group had a possibly high acceptance rate for genome editing in this survey. However, the Pt and GA groups showed high concerns for germline genome editing regardless of awareness (Fig. 2). In the Pt group, respondents who indicated “understanding what it means” had concerns about humans changing the genes of other humans (*P* < 0.01). This result indicated that Pts anticipate the application of genome editing, but they have ambivalent feelings, similar to GAs.

**Fig. 1:**
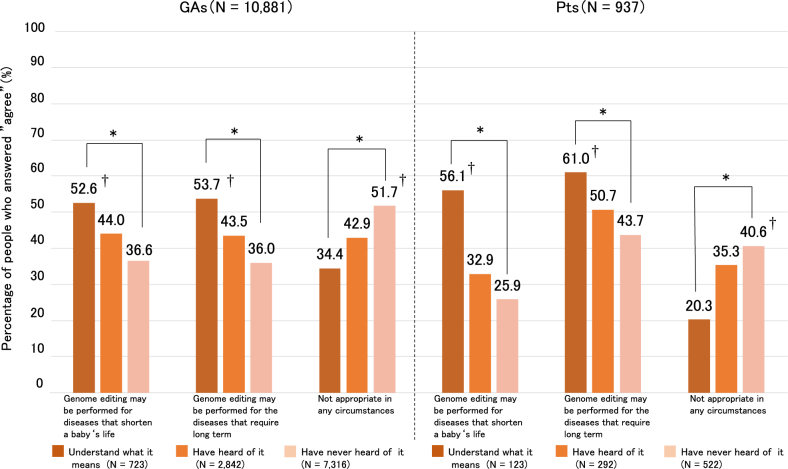
Relationship between awareness and acceptance rate of germline genome editing. A chi-squared test of independence was performed, and a residual analysis was applied when significant results were observed. GAs general adults, Pts patients with disease conditions related to their genetic makeup. * Indicates statistical significance (*P* < 0.01). ^†^Indicates significant at the residual analysis (adjusted normalized absolute value of the residual >1.96; *P* > 0.05)

**Fig. 2:**
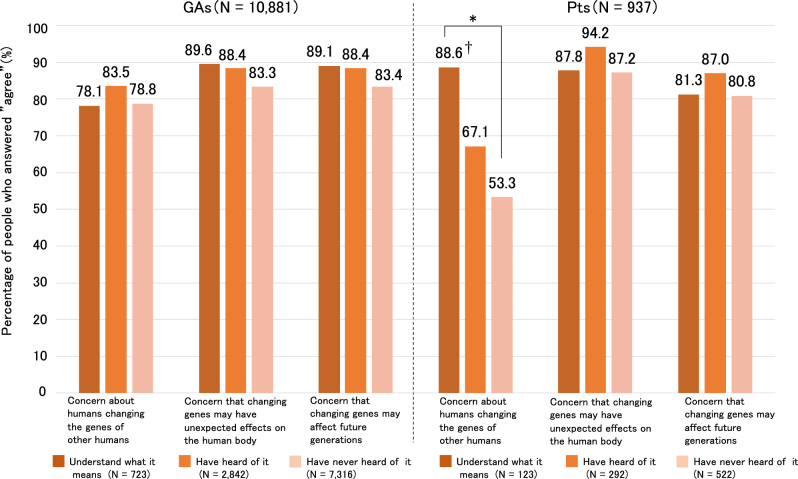
Relationships between awareness and perceived risks of human germline genome editing. A chi-squared test of independence was performed, and a residual analysis was applied when significant results were observed. GAs general adults, Pts patients with disease conditions related to their genetic makeup. ^*^ Indicates statistical significance (*P* < 0.01). ^†^ Indicates significant at the residual analysis (adjusted normalized absolute value of the residual >1.96; *P* > 0.05)

This survey had a potential recruitment bias because it was conducted online and the health conditions of Pts were self-reported. Moreover, it is also thought that detailed investigation on awareness is necessary. However, the results provided insights into the attitude of the public toward the use of genome editing in Japan. Despite low awareness and inadequate understanding about genome editing before responding to our survey, our respondents were accepting of its use in targeting of disease-related genes, albeit with substantial concerns about risks.

This survey also showed that Pts anticipate the application of genome editing, although with substantial concerns about risks. The desire of Pts to avoid transferring their pathogenic gene mutations to future generations may be a reason for these differences. Therefore, we think that increasing awareness of genome editing is necessary, and science communicators and scientists should involve such vulnerable stakeholders in discussion on the topic, while considering and referencing ethical and other associated socio-scientific issues [9].

As inclusivity is an important factor in responsible science [10], appropriate forums should be established where all interested stakeholders can participate. Open and fact-based discussions will allow participants to have more concrete ideas. However, science communicators and scientists must direct attention to the whole process of discussions and be careful not to harm or stigmatize vulnerable participants. Although preimplantation genetic diagnosis (PGD) was not investigated here, we believe that a conscious survey, including that for the use of PGD, should be conducted.

Soon, awareness of germline genome editing is predicted to increase owing to media exposure. Therefore, it is important to continuously conduct similar investigations to track changes in the acceptance of genome editing and perception of its risks in the public.
